# Dynamic parameters for fluid responsiveness in mechanically ventilated children: A systematic review

**DOI:** 10.3389/fped.2022.1010600

**Published:** 2022-10-21

**Authors:** Patcha Yenjabog, Wacharoot Kanchongkittiphon, Somchai Chutipongtanate, Rojjanee Lertbunrian, Patompong Ungprasert

**Affiliations:** ^1^ Division of Pediatric Critical Care, Department of Pediatrics, Faculty of Medicine Ramathibodi Hospital, Mahidol University, Bangkok, Thailand; ^2^ Division of Allergy and Immunology, Department of Pediatrics, Faculty of Medicine Ramathibodi Hospital, Mahidol University, Bangkok, Thailand; ^3^ Pediatric Translational Research Unit, Department of Pediatrics, Faculty of Medicine Ramathibodi Hospital, Mahidol University, Bangkok, Thailand; ^4^ Department of Clinical Epidemiology and Biostatistics, Department of Pediatrics, Faculty of Medicine Ramathibodi Hospital, Mahidol University, Bangkok, Thailand; ^5^ Chakri Naruebodindra Medical Institute, Faculty of Medicine Ramathibodi Hospital, Mahidol University, Samut Prakan, Thailand; ^6^ Department of Rheumatic and Immunologic Diseases, Cleveland Clinic, Cleveland, OH, United States

**Keywords:** fluid therapy, cardiac output, predict, pediatric, hemodynamic

## Abstract

**Objective:**

Fluid administration is the initial step of treatment of unstable pediatric patients. Evaluation of fluid responsiveness is crucial in mechanically ventilated children to avoid fluid overload, which increases mortality. We aim to review and compare the diagnostic performance of dynamically hemodynamic parameters for predicting fluid responsiveness in mechanically ventilated children.

**Design:**

A systematic review was performed using four electronic databases, including PubMed, EMBASE, Scopus, and Central, for published articles from 1 January 2010 to 31 December 2020. Studies were included if they described diagnostic performance of dynamic parameters after fluid challenge was performed in mechanically ventilated children.

**Settings:**

Pediatric intensive and cardiac intensive care unit, and operative room.

**Patients:**

Children aged 1 month to 18 years old who were under mechanical ventilation and required an intravenous fluid challenge.

**Measurements and Main Results:**

Twenty-seven studies were included in the systematic review, which included 1,005 participants and 1,138 fluid challenges. Respiratory variation in aortic peak velocity was reliable among dynamic parameters for predicting fluid responsiveness in mechanically ventilated children. All studies of respiratory variation in aortic peak velocity showed that the area under the receiver operating characteristic curve ranged from 0.71 to 1.00, and the cutoff value for determining fluid responsiveness ranged from 7% to 20%. Dynamic parameters based on arterial blood pressure (pulse pressure variation and stroke volume variation) were also used in children undergoing congenital heart surgery. The plethysmography variability index was used in children undergoing neurological and general surgery, including the pediatric intensive care patients.

**Conclusions:**

The respiratory variation in aortic peak velocity exhibited a promising diagnostic performance across all populations in predicting fluid responsiveness in mechanically ventilated children. High sensitivity is advantageous in non-cardiac surgical patients and the pediatric intensive care unit because early fluid resuscitation improves survival in these patients. Furthermore, high specificity is beneficial in congenital heart surgery because fluid overload is particularly detrimental in this group of patients.

**Systematic Review Registration:**

https://www.crd.york.ac.uk/prospero/display_record.php?RecordID=206400

## Introduction

Fluid administration is the first line of treatment for critically ill children who are admitted to the pediatric intensive care unit (PICU) with unstable hemodynamics. However, only 40% to 69% of these children show a response to fluid administration (1). Fluid responsiveness is defined as an increase in cardiac output of more than 10% to 15% after an intravenous fluid challenge (1–3). Early administration of fluid in patients who are responsive improves survival. However, fluid administration to those who are unresponsive may cause fluid overload, leading to longer ventilator days and higher morbidity and mortality rates (4–6).

Many hemodynamic parameters have been used to predict fluid responsiveness in critically ill children. These parameters can be divided into static and dynamic parameters (Supplementary Table S1). Static parameters are measured at a specific time point during observation. Dynamic parameters are measured by monitoring changes in physiological responses based on cardiopulmonary interaction (e.g., variability change in preload during mechanical ventilation). Most studies have suggested that dynamic parameters are more accurate than static parameters for predicting fluid responsiveness (1, 7–9).

Dynamic parameters can be measured in an invasive or non-invasive manner. Ultrasonic cardiac output monitoring and electrical cardiometry are non-invasive methods that are commonly used to assess dynamic parameters in the intensive care unit (ICU) setting.

Previous studies of dynamic parameters were conducted in different circumstances and populations (10–36). To date, there are no standard parameters that can be used across all critically ill children, especially in mechanically ventilated children, who are prone to fluid overload. This systematic review aimed to compare the diagnostic performance of dynamic parameters for predicting fluid responsiveness in mechanically ventilated children.

## Materials and methods

This study followed the Preferred Reporting Items for Systematic Reviews and Meta-Analysis (PRISMA) reporting guideline (37). The protocol was registered and approved by the international prospective register of systematic reviews PROSPERO (CRD42020206400) on 1 October 2020. Inclusion criteria included the following: (i) children aged 1 month to 18 years old who were under mechanical ventilation and required an intravenous fluid challenge; (ii) diagnostic accuracy studies of dynamic parameters for predicting fluid responsiveness compared with the gold standard definition of fluid responsiveness (10%–15% increase in cardiac output after a fluid challenge as measured by the pressure recording analytic method, an echocardiogram, or non-invasive cardiac output monitoring), and the measurements needed to be performed before and after a fluid challenge; and (iii) the diagnostic performance included the cutoff value, sensitivity, specificity, and area under the receiver operating characteristic (ROC) curve. Meta-analyses, systematic reviews, narrative reviews, clinical practice guidelines, conference proceedings, case series and case reports with a sample size < 10, and non-English articles were excluded.

### Outcome

The primary outcome was to study the diagnostic performance of dynamic hemodynamic parameters, including sensitivity, specificity, and the area under ROC curve, for the prediction of fluid responsiveness in mechanically ventilated children. The secondary outcome was to identify the reliable dynamic parameters among mechanically ventilated children in different clinical circumstances.

### Search strategy

A systematic review was performed using four electronic databases, including PubMed, EMBASE, Scopus, and Central, for published articles from 1 January 2010 to 31 December 2020. The last search was conducted on 15 January 2021. The search terms were *fluid, volume, response, challenge, bolus, and guided*. These words were combined with the medical subject heading (MeSH) terms *hemodynamics, hemodynamic monitoring, fluid therapy, cardiac output, infant, child, adolescent, and pediatrics*. An additional search for potentially eligible articles was carried out using references of selected retrieved articles.

### Study selection and risk of bias assessment

Two authors (P.Y. and W.K.) independently reviewed abstracts of the retrieved articles for their eligibility. Articles that clearly did not fulfill the inclusion criteria were excluded at this stage. The remaining articles underwent a full-text review for final determination of their eligibility Any disagreements were resolved by conference with a third author (R.L.). The risk of bias was assessed using the Quality Assessment of Studies of Diagnostic Accuracy tool (38, 39), which is composed of the following 4 domains: patient selection, index test, reference standard, and flow-timing, while the applicability concern was assessed through 3 domains: patient selection, index test, and reference standard. The risk of bias and applicability concern was judged as “low”, “high”, or “unclear.” If a study was judged as “low” in all domains relating to bias or applicability, then the overall judgment of a “low risk of bias” was assigned for that study. If a study was judged as “high” in one or more domains, it was judged as a “high risk of bias”. The term “unclear” was assigned only when there were missing data that could not be retrieved.

### Data extraction and data synthesis

Two authors (P.Y. and R.L.) independently extracted data from the included articles using a standardized data extraction form derived from the Cochrane Public Health Group Data Extraction and Assessment Template. We contacted the corresponding author of the included articles for missing data. However, only 2 of 10 corresponding authors replied. Those missing data were labeled as not reported.

The following data were collected for systematic review: sample size, age, specific circumstance of participants, definition and percentage of fluid responsiveness, cutoff value, and diagnostic performance of dynamic parameters.

## Results

**Figure 1:**
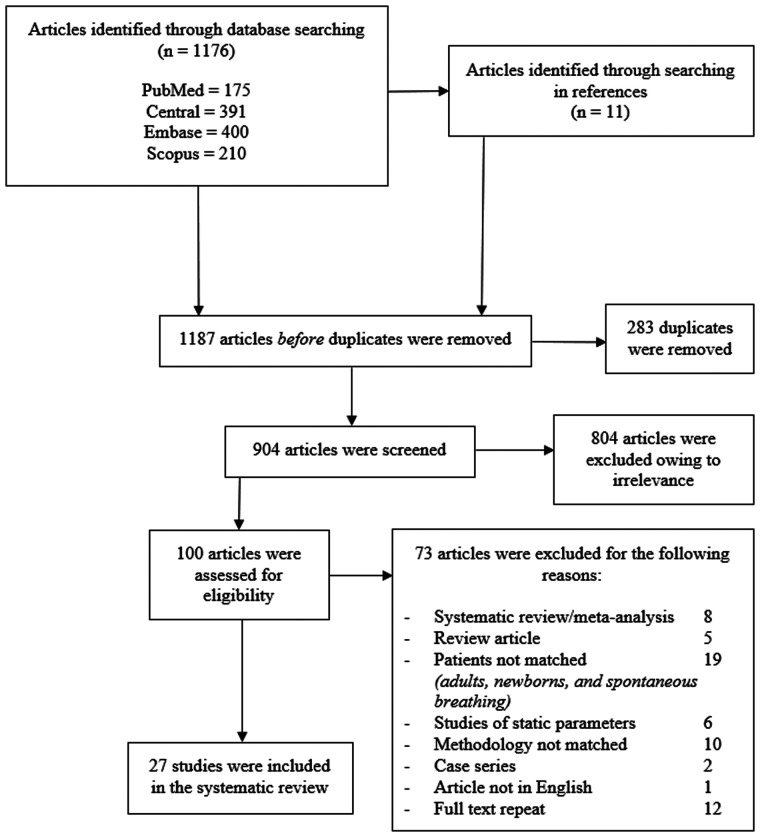
Flowchart of a literature search and study selection.

The identification and selection of studies are shown in Figure 1. A total of 27 studies were included in the final systematic review (10–36), which comprised 1,005 participants and 1,138 intravenous fluid challenges. A total of 77% (21/27) of studies were published after the last systematic review (1). Twenty-five studies were conducted as prospective observational cohorts (10–16, 18–36), and only 1 study was retrospective cohort study (17). There were 4 major groups of patients in different clinical settings as follows: (i) the congenital heart surgery group in 14 studies; (ii) the general surgery group in 5 studies; (iii) the neurological surgery group in 4 studies; and (iv) the general PICU group in 4 studies. Among the subgroups of participants, different fluid types and volumes were administered. Patients with congenital heart surgery mostly received colloid or blood components; only 2 of 14 studies used isotonic crystalloids. The other 3 groups of participants mostly received crystalloids with larger bolus volumes.

Table 1 shows the diagnostic performance of dynamic parameters compared with the gold standard measurement of fluid responsiveness. The gold standard measurement was an increase in cardiac output of 10%–15% after fluid administration, which was represented by multiple parameters as follows: the stroke volume index in 15 studies, stroke volume in 5 studies, the cardiac index in 4 studies, and the velocity–time integral in 2 studies. Eleven dynamic parameters (see Supplementary Table S2 with equations) were investigated in the 27 included studies.

**Table 1:** Characteristics of included studies.

Author, year	Sample size	Age	Setting/population	Fluid type/ volume (ml/kg)	Fluid responder	Parameters/measurement tools	Cutoff value (%)	Sensitivity (%)	Specificity (%)	AUROC curve	Measurement of fluid responsiveness
Choi et al., 2010 (10)	21	Mean: 30 months	Cardiac surgery (after VSD repair)	10 ml/kg 6% HES	11/21 (52%)	ΔVpeak (aortic) TTE	20	91	90	0.830	ΔSV ≥ 15%, TTE
Renner et al., 2011 (11)	27	Mean: 17 months	Congenital heart disease (before surgery of single/ biventricular repair)	10 ml/kg 6% HES	13/27 (48%)	ΔVpeak (aortic) TEE	7	100	84	0.920	ΔSVI ≥ 15%, TEE
						ΔVTI (aortic) TEE	4	84	76	0.840	
						PVI Pulse oximeter	13	84	61	0.780	
Renner et al., 2012 (12)	26	4–48 months	Cardiac surgery (before VSD/ASD repair)	10 ml/kg 6% HES	15/26 (58%)	PPV PRAM	16	61	96	0.790	ΔSVI ≥ 15%, TEE
						SVV PRAM	14	NR	NR	0.700	
			Cardiac surgery (after VSD/ASD repair)			PPV PRAM	15	93	72	0.860	
						SVV PRAM	15	60	90	0.780	
Lee et al., 2014 (13)	26	Mean: 28 months	Cardiac surgery (after VSD repair)	10 ml/kg 6% HES	13/26 (50%)	SVV NICOM®	10	77	85	0.888	ΔSV ≥ 15%, TEE
						ΔVpeak (aortic) TEE	14	85	92	0.956	
Saxena et al., 2015 (14)	100	Median: 18 months	Cardiac surgery (n = 90) Others (n = 10)	10 ml/kg Isotonic crystalloid	64/142 (45%)	SPV PRAM	NR	NR	NR	0.590	ΔSVI ≥ 15%, TPUD
						PPV PRAM	NR	NR	NR	0.540	
						SVV PRAM	NR	NR	NR	0.530	
Lee et al., 2015 (15)	29	1–36 months	Cardiac surgery (after ASD/VSD/TOF/AVSD repair)	10 ml/kg 6% HES	13/29 (45%)	SVV NICOM®	NR	NR	NR	0.510	ΔSVI > 15%, TEE
						ΔVpeak (aortic) TEE	13.5	69.2	78.6	0.770	
Han et al., 2017 (16)	38	Mean: 1.05 years	Cardiac surgery (after VSD repair)	20 ml/kg 5% albumin or FFP	27/38 (71%)	PPV PRAM	17.4	89	91	0.890	ΔCI ≥ 15%, PRAM
	36	Mean: 1.15 years	Cardiac surgery (after TOF repair)		26/36 (72%)	PPV PRAM	13.4	81	80	0.790	
Favia et al., 2017 (17)	16	NR	Cardiac surgery (after CHD repair of biventricular physiology)	10 ml/kg crystalloid or blood component	7/16 (44%)	PPV PRAM	30	67	100	0.760	ΔCI ≥ 10%, TEE
						ΔVTI TEE	17	83	77	0.760	
Lee et al., 2017 (18)	30	Mean: 19 months	Cardiac surgery (after VSD/ASD repair)	10 ml/kg 6% HES	17/30 (57%)	Calibrated abdominal compression of 30 mmHg for 15 s PRAM for ΔDBP	5	82.4	69.3	0.778	ΔSVI > 15%, TEE
						ΔVpeak (aortic) TEE	12	58.8	84.6	0.765	
Han et al., 2017 (19)	26	3–12 months	Cardiac surgery (VSD repair) Median sternotomy group	16 ml/kg 5% albumin or blood components	12/26 (46%)	PPV PRAM	19	92	71	0.850	ΔCI ≥ 15%, PRAM
	29		Cardiac surgery (VSD repair) Right thoracotomy group		16/29 (55%)	PPV PRAM	18	94	69	0.830	
Cheng et al., 2018 (20)	60	Mean 10.9 months	Cardiac surgery (after VSD/ASD/PDA repair)	10 ml/kg 6% HES	32/60 (53%)	SVV USCOM®	17	84.4	60.7	0.776	ΔSVI ≥ 15%, USCOM®
Kim et al., 2019 (21)	30	1–12 months	Cardiac surgery (after VSD/ASD repair)	10 ml/kg isotonic crystalloid	17/30 (57%)	ΔVpeak (carotid) Doppler US	7.8	94	69	0.830	ΔSVI > 15%, TEE
						ΔVpeak (aortic) TEE	13	77	92	0.860	
Park et al., 2019 (22)	38	1–6 months 0	Cardiac surgery (after VSD/ASD repair) and neurosurgery	10 ml/kg 6% HES	20/38 (53%)	ΔPOP at 0.9–1.2 N contraction force Pulse oximetry	15	NR	NR	0.815	ΔSVI > 15%, TEE/TTE
						ΔPOP with individual adjustment for contraction force Pulse oximetry	11	NR	NR	0.847	
Song et al., 2020 (23)	64	3–8 years	Cardiac surgery (after the Fontan operation with fenestration)	10 ml/kg 5% albumin	30/64 (47%)	SVV PRAM	16	50	91.7	0.740	ΔCI ≥ 15%, PRAM
Julien et al., 2013 (24)	54	Median: 48 months	General surgery	10 ml/kg isotonic crystalloid	45/97 (46%)	PVI Pulse oximeter	13	80	80	0.850	ΔSVI > 15%, CardioQ®
Achar et al., 2016 (25)	42	12–168 months	General elective surgery (preoperative)	10 ml/kg balanced salt solution	24/42 (57%)	ΔVpeak (aortic) TTE	12.2	100	94.4	0.975	ΔSVI > 15%, TTE
						IVC-DI US	23.5	91	89	0.940	
Kim et al., 2020 (26)	30	10–72 months	General procedure (under general anesthesia)	10 ml/kg isotonic crystalloid	17/30 (57%)	PVI Transflectance adhesive forehead sensor	6	94.1	61.5	0.800	ΔSVI > 15%, TTE
						PVI Finger sensor	9	64.7	76.9	0.700	
						ΔVpeak (aortic) TTE	10.6	94.1	61.5	0.800	
Chen et al., 2020 (27)	27	8 months to 13 years	Liver cirrhosis (during liver transplantation)	10 ml/kg isotonic crystalloid	15/61 (25%)	PPV PRAM	13	46.7	80.4	0.670	ΔSVI ≥ 15%, TPUD
						SVV PRAM	10	80	54.4	0.680	
						PVI Pulse oximeter	NR	NR	NR	0.560	
Zorio et al., 2020 (28)	55	6–148 months	General elective Surgery	12 ml/kg isotonic crystalloid/balanced salt solution	43/55 (78%)	Mini-fluid bolus (3 ml/kg in 2 min) TTE for ΔVTI	8	53	77	0.770	ΔVTI ≥ 10%, TTE
Pereira de Souza Neto et al., 2011 (29)	19	5.5–71 months	Neurological surgery (craniosynostosis and posterior fossa tumor)	20 ml/kg isotonic crystalloid	10/19 (53%)	ΔVpeak (aortic) TTE	10	100	100	1.000	ΔVTI ≥ 15%, TTE
						ΔPP/PPV PRAM	NR	NR	NR	0.710/ 0.630	
						ΔPOP/PVI Pulse oximeter	NR	NR	NR	0.510/ 0.630	
	11	72–143 months	Neurological surgery (posterior fossa tumor)	20 ml/kg isotonic crystalloid	7/11 (64%)	ΔVpeak (aortic) TTE	10	100	100	1.000	
						ΔPP/PPV PRAM	NR	NR	NR	0.600/ 0.600	
						ΔPOP/PVI Pulse oximeter	NR	NR	NR	0.570/ 0.540	
Byon et al., 2013 (30)	33	6–108 months	Neurological surgery (during surgery)	10 ml/kg 6% HES or Voluven	15/33 (45%)	PVI Pulse oximeter	11	73	87	0.767	ΔSVI ≥ 10%, TTE
						ΔVpeak (aortic) TTE	11	87	72	0.804	
Vergnaud et al., 2015 (31)	30	4–139 months	Neurological surgery (after craniosynostosis repair)	20 ml/kg Artificial colloid	15/30 (50%)	PPV NICOM®	8	69	78	0.770	ΔSV ≥ 15%, TTE
						SVV NICOM®	10	80	93	0.810	
Morparia et al., 2018 (32)	21	28 months to 17 years	Elective neurological Surgery	10 ml/kg isotonic crystalloid	13/22 (59%)	ΔVpeak (aortic) TTE	12.3	77	89	0.902	ΔSV > 15%, TTE
McLean et al., 2014 (33)	13	2–168 months	General PICU	10 ml/kg isotonic crystalloid	11/26 (42%)	SVV USCOM®	16.5	54.5	93.3	0.797	ΔSVI ≥ 10%, USCOM®
Weber et al., 2015 (34)	31	Median: 36 months	General PICU	10 ml/kg isotonic crystalloid	15/31 (48%)	SVV PRAM (LiDCO_rapid_)	NR	NR	NR	0.513	ΔSVI > 10%, TTE
						IVC-DI US	NR	NR	NR	0.502	
Chaiyaphruk et al., 2018 (35)	13	3 months to 15 years	General PICU	5–10 ml/kg isotonic crystalloid	6/13 (46%)	PLR 45° for 2 min USCOM® for ΔCI	8	60	83.3	NR	ΔCI ≥ 10%, USCOM®
Sun et al., 2020 (36)	30	1 month to 18 years	Leukemia with neutropenia and septic shock	20 ml/kg isotonic crystalloid	16/30 (53%)	ΔVpeak (aortic) TTE	12.4	62	64	0.710	ΔSV ≥ 15%, TTE
						ΔVTI (aortic) TTE	13.7	81	79	0.740	

Abbreviations: ASD, atrial septal defect; AUROC, area under the receiver operating characteristic; AVSD, atrioventricular septal defect; CHD, congenital heart disease; CI, cardiac index; DBP, diastolic blood pressure; FFP, fresh frozen plasma; HES, hydroxyethyl starch; IVC-DI, inferior vena cava distensibility index; LIDCOrapid, a pulse contour analysis algorithm system; N, Newton; NICOM, non-invasive cardiac output monitoring; NR, not reported; PDA, patent ductus arteriosus; PICU, pediatric intensive care unit; PLR, passive leg raising test; PRAM, pressure recording analytic method; PVI, plethysmographic variability index; SV, stroke volume; SVI, stroke volume index; SVV, stroke volume variation; TEE, transesophageal echocardiogram; TOF, tetralogy of Fallot; TPUD, transpulmonary ultrasound dilution; TTE, transthoracic echocardiogram; US, ultrasound; USCOM, ultrasonic cardiac output monitoring; VSD, ventricular septal defect; ΔVpeak, respiratory variation in aortic peak velocity; VTI, velocity–time integral.

The respiratory variation in aortic peak velocity (ΔVpeak) was the most common dynamic parameter examined (12/27 studies). Moreover, ΔVpeak provided a reliable diagnostic performance. All studies of ΔVpeak showed that the area under the ROC curve ranged from 0.71 to 1.00, and the cutoff value of ΔVpeak for determining fluid responsiveness ranged from 7% to 20%.

Because patients with congenital heart surgery were included in approximately half of all studies, we allocated participants to 2 new subgroups as follows: the congenital heart surgery subgroup (10–23) and the non-cardiac surgery subgroup (general surgery, neurological surgery, and general PICU patients) (24–36). In congenital heart surgery subgroup, ΔVpeak showed the best sensitivity of 100% at the cutoff value of 7% when performed by transesophageal echocardiogram (TEE) (11). The best specificity of ΔVpeak was 92% at the cutoff values 13%–14% by TEE (13, 21). Another reliable dynamic was the pulse pressure variation (PPV), with the sensitivity of 94% (at the cutoff value of 18%) and the specificity of 100% (at the cutoff value of 30%) (17). In the non-cardiac surgery subgroup, ΔVpeak performed by transthoracic echocardiogram (TTE) showed the best sensitivity of 100% (at the cutoff values 10% and 12.2%) (25, 29) with the best specificity of of 100% (at the cutoff value 10%) (29). Note that plethysmographic variability index (PVI) measured by the transflectance adhesive forehead sensor exhibited the second-best sensitivity of 94.1% (at the cutoff value of 6%) (26), while stroke volume variation (SVV) provided the second-best specificity of 93.3% (at cutoff values 16.5%) (33).

The risk of bias assessment of all included studies is shown in Table 2. The reference standard domain was judged to have a high risk of bias in 9 studies because the interpretation of the reference standard test was made with knowledge of index test results. The flow and timing domain were also judged to have a high risk of bias in 15 studies because all included patients were not in the final analysis (per-protocol analysis).

**Table 2:** Risk of bias assessment.

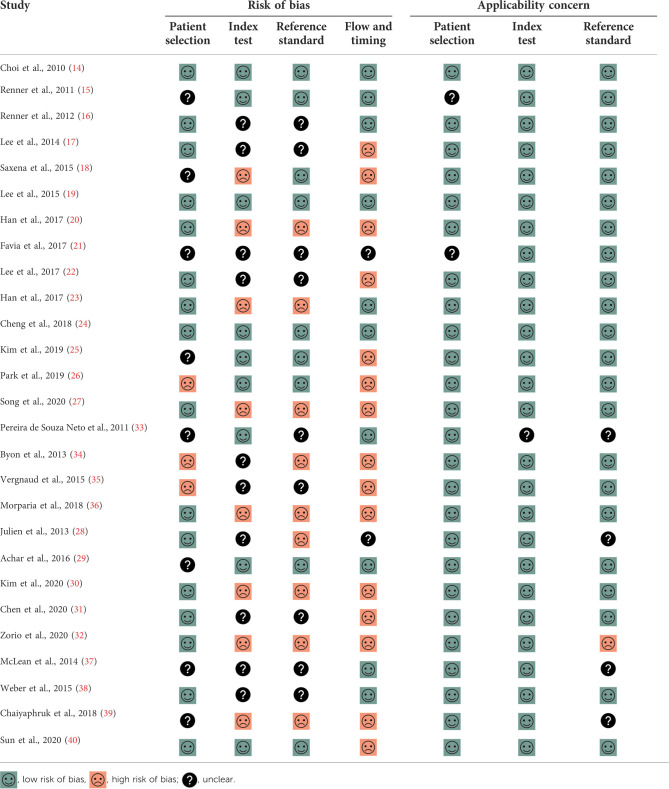

## Discussion

In 2013, Gan et al. (1) studied static and dynamic parameters, and found that dynamic parameters were more reliable in predicting fluid responsiveness in children. Several new dynamic parameters have since been introduced and studied in the pediatric population during the last 10 years. Therefore, we conducted this review to extend the work of Gan et al. (1) on dynamic parameters and to provide an update with newly examined parameters.

New dynamic parameters from non-invasive ultrasonic cardiac output monitoring, electrical cardiometry, and ultrasound are easily accessible and widely used in the PICU. These new parameters are reliable and can be measured by non-experienced physicians in a few minutes (40, 41). Therefore, they could be useful tools for clinicians to determine whether patients should undergo a fluid challenge.

This systematic review showed that ΔVpeak had a promising diagnostic performance across all populations. The ΔVpeak was studied as a single parameter or together with other dynamic parameters. The cutoff values for predicting fluid responsiveness ranged from 7% to 20%, while the average values ranged from 12% to 14%. In group of congenital heart surgery, the echocardiogram performed by transesophageal technique but in other groups, mostly performed by transthoracic technique. A major disadvantage of ΔVpeak is that this parameter requires an experienced operator of echocardiography.

The highest sensitivity of ΔVpeak in patients who had congenital heart surgery is advantage because fluid overload can increase the risk of acute kidney injury and poor postoperative outcomes in patients with congenital heart disease (42, 43). Therefore, a parameter with high specificity, such as ΔVpeak, could reduce such adverse events and complications by decreasing an unnecessary fluid challenge in this patient subgroup. When ΔVpeak is not accessible, new dynamic parameters from non-invasive methods such as ultrasonic cardiac output monitoring, electrical cardiometry, and arterial line variable parameters should be considered, because of easy accessibility and mostly non-operator dependent methods. Pulse pressure variation could be used as alternative because it also had a high specificity. Patients in the non-cardiac subgroup are most likely to benefit from early fluid resuscitation. The ΔVpeak and PVI should be considered in this context because they have a high sensitivity.

Each study with patients in the congenital heart surgery group reported inotropic and vasopressor administration in various forms, including the percentage of inotrope use in the population and the Vasoactive Inotropic Score, and some studies did not report inotropic or vasopressor data. Therefore, we did not perform analysis for specific dynamic parameters based on inotropic status.

There are some limitations to our study. First, our search strategy was limited to the last 10 years. The reason for his limitation was to focus on new dynamic parameters that appeared after the systematic review in 2013 by Gan et al. (1) Second, there was heterogeneity of the study design, including multiple participant groups in different clinical settings, different fluid types, varying amounts of volume (5–20 ml/kg), and the definition of fluid responsiveness using different parameters across the studies.

The findings from this systematic review suggest some future research opportunities. The ΔVpeak, which is the most reliable parameter for predicting fluid responsiveness in mechanically ventilated children, has not been investigated in children with spontaneous breathing. Preload challenge maneuvers (e.g., calibrated abdominal compression, mini-fluid bolus, the passive leg raising test, and the end-expiratory occlusion test) have been extensively studied in the adult population for predicting fluid responsiveness (44). However, these maneuvers have not been well investigated in pediatric population.

## Conclusions

The ΔVpeak exhibited a promising diagnostic performance in predicting fluid responsiveness in mechanically ventilated children. The sensitivity of ΔVpeak is advantageous in non-cardiac surgical patients and the PICU setting because early fluid resuscitation improves survival in these patients. Furthermore, the specificity of ΔVpeak is beneficial in congenital heart surgery because fluid overload is particularly detrimental in this group of patients.

## Data Availability

The raw data supporting the conclusions of this article will be made available by the authors, without undue reservation.
