# Supplementary material for: Dynamic parameters for fluid responsiveness in mechanically ventilated children: A systematic review
Source: Front Pediatr. 2022 Oct 21;10:1010600. doi: 10.3389/fped.2022.1010600 (PMC9638161; doi:10.3389/fped.2022.1010600)
Supplement: Supplementary file 1 [file Table1.docx]

**eTable 1. Static and dynamic parameters for predicting fluid responsiveness.**

| Static parameters | Dynamic parameters |
| --- | --- |
| Clinical examination   - Heart rate - Blood pressure | **Arterial pressure**   - Systolic pressure variation - Pulse pressure variation - Stroke volume variation |
| Preload pressure   - Central venous pressure - Pulmonary artery occlusion pressure | **Plethysmography**   - Plethysmographic amplitude variation - Plethysmographic variability index |
| Thermodilution and ultrasound dilution   - Global end-diastolic volume index - End-diastolic volume - Ejection fraction | **Cardiac preload challenge**   - Abdominal compression - Passive leg rising test - End-expiratory occlusion test - Mini-fluid challenge test |
| Echocardiography and Doppler   - Left ventricular end-diastolic area - Stroke volume index - Corrected flow time | **Echocardiography and Doppler**   - Respiratory variation in aortic blood flow peak velocity - Respiratory variation in the inferior vena cava diameter - Stroke distance variation - Velocity–time integral |
