# Supplementary material for: Dynamic parameters for fluid responsiveness in mechanically ventilated children: A systematic review
Source: Front Pediatr. 2022 Oct 21;10:1010600. doi: 10.3389/fped.2022.1010600 (PMC9638161; doi:10.3389/fped.2022.1010600)
Supplement: Supplementary file 2 [file Table2.docx]

**eTable 2. List of dynamic parameters in all included studies.**

| Dynamic parameters | Formula |
| --- | --- |
| Systolic pressure variation  (%) | $\frac{\left( SBPmax-SBPmin \right)\times100}{SBPmean}$ |
| PP variation  (%) | $\frac{\left( PPmax-PPmin \right)\times100}{PPmean}$ |
| SV variation  (%) | $\frac{\left( SVmax-SVmin \right)\times100}{SVmean}$ |
| Plethysmographic variability index  (%) | $\frac{\left( PImax-PImin \right)\times100}{PImax}$ |
| Plethysmographic amplitude variation  (%) | $\frac{\left( AMPmax-AMPmin \right)\times100}{AMPmean}$ |
| Respiratory variation in aortic peak velocity (%) | $\frac{\left( VPeakmax-VPeakmin \right)\times100}{VPeakmean}$ |
| VTI  (ΔVTI%) | $\frac{\left( VTImax-VTImin \right)\times100}{VTImean}$ |
| IVC-DI | $\frac{\left( IVCDmax-IVCDmin \right)\times100}{IVCDmin}$ |
| Calibrated abdominal compression was 30 mmHg for 15 s. | |
| A mini-fluid bolus was 3 ml/kg in 2 min. | |
| The passive leg raising test was at 45^o^ for 2 min. | |

Abbreviations: AMP, amplitude of a photoplethysmogram; IVC-DI, inferior vena cava-distensibility index; IVCD, inferior vena cava diameter; max, maximum; min, minimum; PI, perfusion index; PP, pulse pressure; SBP, systolic blood pressure; SV, stroke volume; VPeak, aortic blood flow peak velocity; VTI, velocity time integral.
